# Management of a Female Patient With Traumatic Carotid Aneurysm and Depression

**DOI:** 10.7759/cureus.100964

**Published:** 2026-01-06

**Authors:** Konstantinos Kontoangelos, Michail Tsotsios, Sofia Tsiori, Antonia Skotsimara, Ilias Avgerinos, Natasha Hasemaki, Christos Klonaris

**Affiliations:** 1 First Department of Psychiatry, Eginition Hospital, National and Kapodistrian University of Athens, Athens, GRC; 2 Second Department of Vascular Surgery, School of Medicine, National and Kapodistrian University of Athens, General Hospital of Athens “Laiko”, Athens, GRC

**Keywords:** carotid aneurysm, depression, neuropyschiatry, pain, trauma

## Abstract

Extracranial carotid aneurysms are rare and may be asymptomatic for long periods, or they may present with symptoms depending on their size and location. Complications such as rupture or stroke can significantly impact outcomes, particularly if the aneurysm is not detected early or treated promptly. This case report illustrates a traumatic carotid aneurysm in a young female patient and its surgical management with successful stent-graft deployment. Patients with undiagnosed intracerebral aneurysms that have not ruptured may present with a variety of neurological and psychiatric symptoms. Management of psychiatric conditions in these patients after surgical aneurysm repair is essential, as it improves quality of life.

## Introduction

Carotid artery aneurysms are reported to be rare in the literature and may be sequelae of various etiologies. Both true aneurysms, which involve all layers of the arterial wall, and pseudoaneurysms, which result from disruption of the arterial wall and consist only of the adventitia, can occur [1]. Extracranial carotid aneurysms account for approximately 1% of all arterial aneurysms. Diagnosis may be incidental in asymptomatic cases, or patients may present with a variety of clinical signs, including neurologic symptoms, a pulsatile mass in the neck or oral cavity, mass effect, or excessive bleeding caused by rupture [2].

The common carotid artery originates in the chest from either the innominate artery or the aortic arch and bifurcates into the internal and external carotid arteries. The extracranial portion of the carotid artery extends to the base of the skull, where it enters the carotid canal. True aneurysms are most commonly diagnosed in older individuals and are associated with atherosclerotic disease. Nevertheless, etiologies such as congenital defects, inflammatory diseases, and connective tissue disorders, most notably fibromuscular dysplasia, can predispose individuals to true aneurysm formation [3]. In contrast, trauma, whether blunt or penetrating, is the most common cause of pseudoaneurysms. Infection or prior carotid surgery may also result in pseudoaneurysm formation.

Trauma to the internal carotid artery in the neck can cause localized dissection, which may progress to pseudoaneurysm formation with an associated risk of ischemia or infarction affecting the corresponding cerebral hemisphere [4-6]. Traumatic aneurysms may be located on large basal arteries or distal peripheral branches. Aneurysms arising after surgical procedures such as trephination and tumor removal have also been described. Indirect arterial trauma may occur in severe closed head injuries, in which vessels can be damaged by contact with the falx, tentorium, or bony prominences during significant brain shifts, or may be involved in areas of focal brain necrosis and softening [7]. Major pathophysiological mechanisms of trauma are commonly associated with high-speed motor vehicle collisions and connective tissue disorders. Cervical spine forces such as distraction-extension, distraction-bending, or lateral bending can lead to traumatic carotid aneurysm formation [8].

A traumatic carotid aneurysm can be life-threatening if not treated appropriately, as it may lead to intracranial hemorrhage or other serious complications. In addition, several factors may contribute to the development of depression in patients with traumatic carotid aneurysms, including physical health impacts (neurologic complications, chronic pain, and fatigue), psychological factors (trauma, post-traumatic stress disorder, and fear of future complications), social and emotional factors (disability, reduced independence, and support systems), and biological factors (changes in brain chemistry and medication side effects) [9]. This case report illustrates a traumatic carotid aneurysm in a young female patient and its surgical management with successful stent-graft deployment.

## Case presentation

A 36-year-old female patient presented to the outpatient clinic with a history of long-standing and worsening migraines. The patient reported a road traffic accident seven years earlier, during which she was wearing a seatbelt. She sustained multiple bone fractures that were treated surgically, along with various internal injuries, which caused significant discomfort for an extended period. Although no skull fracture was diagnosed, she gradually developed intense, unbearable headaches radiating to the right temple, accompanied by persistent migraines since the accident.

Five years after the accident, a mass behind the right tonsil was noted, and several months later, severe neck pain developed. An ultrasound examination was performed and revealed an aneurysm arising from the right internal carotid artery.

**Figure 1:**
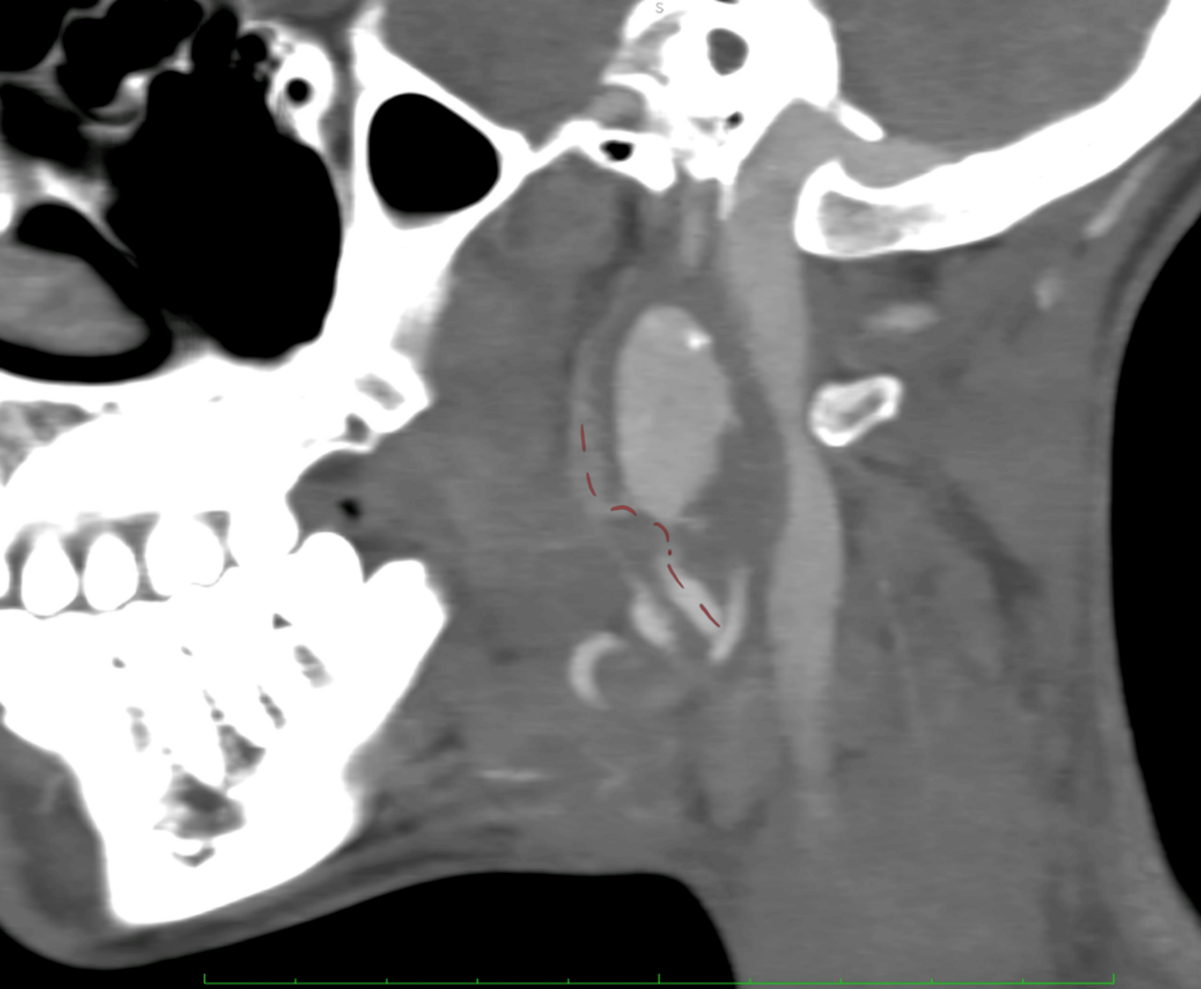
Sagittal view of the pseudoaneurysm arising from the right internal carotid artery The vertebra adjacent to the pseudoaneurysm is the first cervical vertebra.

**Figure 2:**
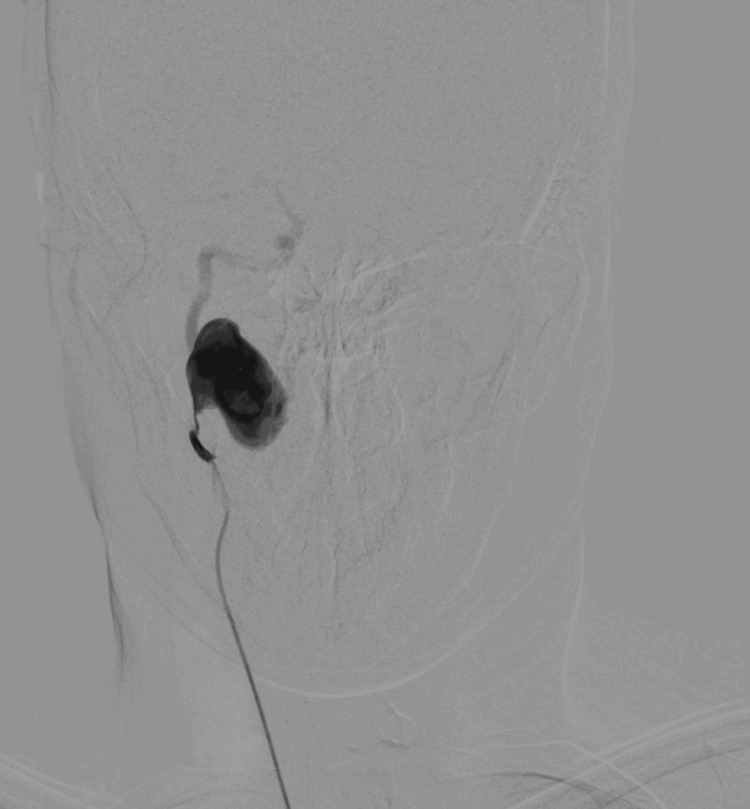
Preoperative DSA demonstrating turbulent flow within the pseudoaneurysm and reduced distal flow DSA, digital subtraction angiography

A CT scan with intravenous contrast was subsequently ordered and demonstrated a large pseudoaneurysm arising from the right internal carotid artery at the level of the first cervical vertebra, with a maximum diameter of 23.7 mm (Figure 1). The right internal carotid artery distal to the aneurysm was significantly smaller compared with the contralateral side. Digital subtraction angiography (DSA) was then performed via percutaneous right femoral artery access under local anesthesia, with selective catheterization of the right carotid artery. This revealed turbulent flow within the aneurysm and reduced flow in the distal carotid artery (Figure 2). These findings were confirmed using transcranial Doppler ultrasound, which demonstrated a reduced velocity of 37 cm/s in the middle cerebral artery.

**Figure 3:**
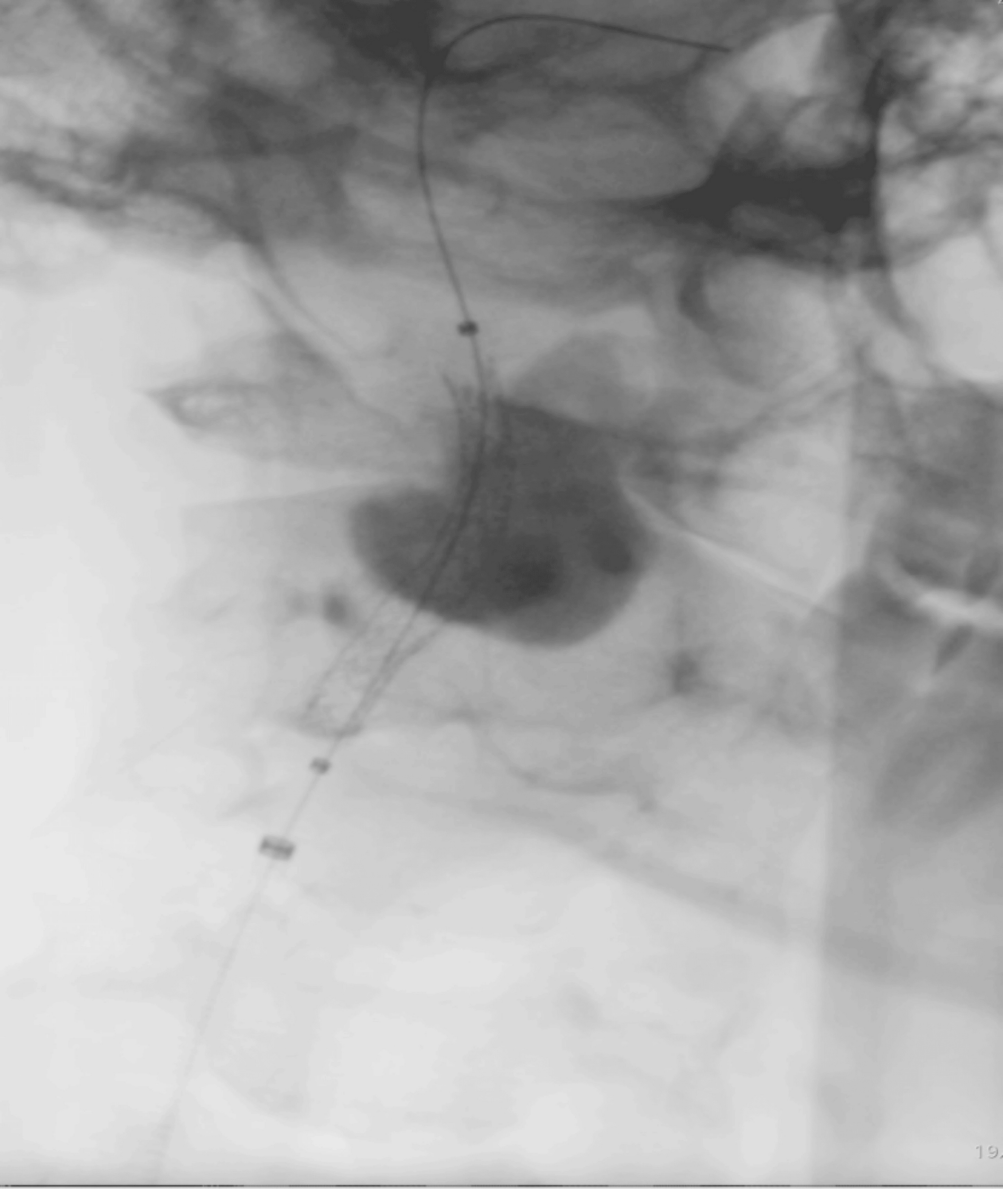
Intraoperative image showing the deployed stent graft with iodinated contrast trapped within the pseudoaneurysm

The patient was admitted to the vascular surgery department, and endovascular repair of the pseudoaneurysm was planned. Under local anesthesia, with continuous neurologic monitoring and percutaneous right femoral access, the right carotid artery was catheterized. After difficult catheterization of the distal segment of the internal carotid artery using a V18 guidewire, a covered stent graft (BeGraft 5 × 38 mm; Bentley InnoMed GmbH, Hechingen, Germany) was deployed, resulting in complete exclusion of the aneurysm and improved flow in the intracranial carotid artery (Figure 3).

The patient remained under observation with no adverse events during the postoperative course. No neurologic signs or symptoms developed, and she was discharged on the second postoperative day on dual antiplatelet therapy with aspirin 100 mg daily and clopidogrel 75 mg daily.

**Figure 4:**
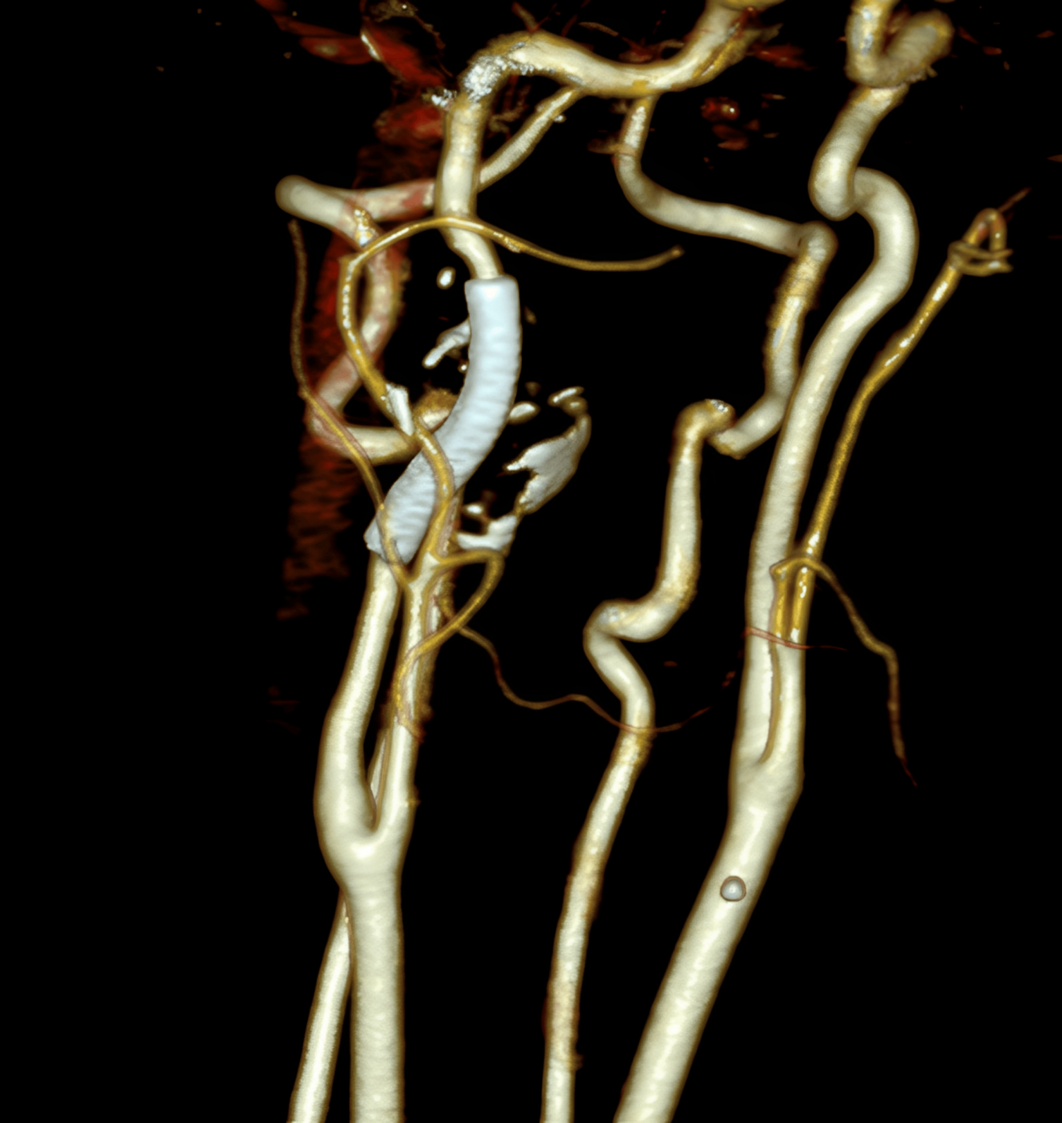
One-year CT angiography reconstruction demonstrating stent-graft patency, complete exclusion of the pseudoaneurysm, and normal distal internal carotid artery diameter

At the six-month follow-up, color Doppler ultrasound demonstrated no blood flow within the aneurysm, with a maximum diameter of 22.7 mm. CT angiography performed one year after the operation demonstrated complete thrombosis of the pseudoaneurysm, with primary patency of the stent graft. The diameter of the distal internal carotid artery was comparable to that of the contralateral side (Figure 4).

During the last year, the patient developed intense depressive symptomatology, including hopelessness about the future, disrupted sleep, fatigue, reduced functionality, and persistent sadness, with mood worsening due to severe migraines. Although her physical recovery progressed satisfactorily, behavioral and emotional changes became evident approximately four weeks after the injury. The patient exhibited persistent symptoms consistent with a depressive disorder, including low mood present most days; a marked reduction in interest and pleasure in previously enjoyed activities; fatigue and reduced energy disproportionate to physical findings; sleep disturbances characterized by prolonged, nonrestorative sleep; decreased appetite with unintentional weight loss; impaired concentration; social withdrawal with reduced verbal engagement; and feelings of hopelessness and reduced self-efficacy.

The patient also experienced severe migraines that further reduced her functionality and contributed to anxiety regarding her professional development. A comprehensive psychiatric evaluation was conducted, including a structured clinical interview, review of neurologic status and current medications, and assessment of psychosocial stressors and family dynamics. The patient met diagnostic criteria for major depressive disorder associated with a medical condition, moderate severity. She received antidepressant treatment with duloxetine 60 mg, resulting in mood improvement. Pharmacotherapy was adapted to accommodate her medical condition and fatigue. The patient and her family received education regarding the relationship between medical trauma and mood disorders, the biological basis of depression, and the importance of early intervention and adherence to treatment.

The patient was initiated on cognitive behavioral therapy, targeting maladaptive cognitive patterns related to illness and recovery, behavioral activation with graded re-engagement in daily activities, coping strategies for uncertainty and health-related anxiety, and emotional processing of traumatic experiences. Over several weeks, the patient demonstrated gradual improvement in mood, energy level, sleep quality, and cognitive functioning. She resumed professional activities with appropriate accommodations and reengaged socially. Ongoing psychiatric follow-up was maintained to ensure sustained recovery.

## Discussion

A series reported 141 carotid aneurysms in 132 patients, of which 82% were pseudoaneurysms, and approximately half were asymptomatic at the time of diagnosis [7]. In the present case, the patient presented with an intraoral pulsatile mass and localized pain associated with migraines. CT or MR angiography is considered necessary in such cases to identify the diameter of the aneurysmal sac and its relationship to the normal artery and surrounding structures, such as the carotid canal [10]. In this case, DSA and transcranial color Doppler were performed to assess the low-flow effect of the aneurysm on cerebral circulation, which appeared to be compromised.

There are no established guidelines for the management of carotid aneurysms, and most available data are derived from single-institution series. In general, all symptomatic aneurysms should be treated, as well as aneurysms larger than 2 cm, those demonstrating rapid expansion, and those with intraluminal thrombus. The choice of treatment is often challenging and depends on individual anatomy, symptomatology, and physician experience. Infected, true, or ruptured aneurysms may be more suitable for open repair, whereas traumatic pseudoaneurysms and hostile neck anatomy may favor an endovascular approach. No randomized controlled trials have compared open and endovascular repair.

Endovascular repair offers several advantages, including a lower risk of cranial nerve and adjacent structure injury, the ability to access more distal lesions, and avoidance of general anesthesia. A limitation of the endovascular approach is the lack of long-term data regarding stent patency in the cervical carotid artery. Available endovascular treatment options include bare-metal stents with or without aneurysm sac embolization, covered stents, carotid artery occlusion, and flow-diverting stents.

In a systematic review by Li et al., 224 patients with carotid artery aneurysms were treated using endovascular techniques, primarily because of distal lesion location or hostile neck anatomy. Covered stents were used in 68% of cases, while distal protection devices were employed in only 2.3%. Primary patency at 15 months was 93.2%. Covered stents appear to offer higher rates of aneurysm sac thrombosis and a reduced risk of reintervention and late complications [11].

Both intracranial and carotid aneurysms have been associated with higher levels of depression and stress compared with the general population. Increased stress levels have been reported in patients with a history of subarachnoid hemorrhage who subsequently present with an unruptured aneurysm [12]. Patients with undiagnosed, unruptured intracerebral aneurysms may present with a range of neurologic and psychiatric symptoms, including migraine without aura, focal or generalized seizures, frontal lobe syndrome, visual impairment, fatigue, and malaise [6]. Intracranial aneurysms of the internal carotid artery may manifest as diplopia, retro-orbital pain, and unilateral headache mimicking migraine without aura. Other intracranial lesions, such as brain tumors and arteriovenous malformations, may also present with neuropsychiatric symptoms. Treatment of the underlying aneurysm may alleviate these manifestations and improve clinical outcomes [13].

Coping mechanisms play a significant role in mental health outcomes following trauma. Individuals with adaptive coping strategies, such as seeking therapy, maintaining social engagement, and fostering resilience, are less likely to develop severe depression. In contrast, social isolation and unaddressed emotional distress may exacerbate depressive symptoms. Management of depression in these patients includes psychological support, pharmacotherapy, support groups, and lifestyle modifications [14]. A multidisciplinary treatment approach involving specialists from multiple fields, such as neurology, psychiatry, and vascular surgery, is essential to address both physical and emotional needs and promote comprehensive recovery [15].

Although direct reports of depression following traumatic carotid aneurysm are limited, cases of intracranial aneurysms presenting primarily with mood disorder symptoms have been described. For example, a pediatric internal carotid artery aneurysm presented with depressive manifestations before neurologic deterioration, and an unruptured anterior communicating artery aneurysm initially manifested as a major depressive episode, with symptom resolution following aneurysm clipping [16].

This case illustrates that depression is a common and clinically significant complication of traumatic carotid aneurysm, even in the absence of overt neurologic deficits. Early recognition, comprehensive assessment, and a multidisciplinary treatment strategy combining psychotherapy, pharmacotherapy, and psychosocial support are essential to optimize outcomes and quality of life. A limitation of this report is that improvement in depressive symptoms was based on clinical psychiatric interviews rather than standardized assessment tools, such as the Patient Health Questionnaire.

## Conclusions

Management of mental health in patients following surgical aneurysm repair is important, as it improves quality of life and reduces symptoms of anxiety and fear associated with the perceived risk of rupture. Aneurysm repair improved the patient’s quality of life, contributed to a reduction in anxiety and migraine frequency, and enhanced professional functioning. Combined surgical treatment of the aneurysm and management of depression with pharmacotherapy and psychotherapy significantly improved the patient’s overall quality of life. Assessment of the patient’s baseline psychological status is important to determine readiness to return to professional and social activities and to identify whether psychological support may be beneficial both before and after surgery.
